# Detection of Lymphadenopathy as a Precursor to Autoimmune Liver Diseases Before Clinical Hepatitis Became Apparent: A Report of Two Cases

**DOI:** 10.7759/cureus.47595

**Published:** 2023-10-24

**Authors:** Hiroshi Okano, Hiroki Tanaka, Shimpei Matsusaki, Katsumi Mukai, Akira Nishimura, Kana Asakawa, Youichirou Baba, Tetsuya Murata

**Affiliations:** 1 Gastroenterology, Suzuka General Hospital, Suzuka, JPN; 2 Pathology, Suzuka General Hospital, Suzuka, JPN

**Keywords:** autoimmune hepatic diseases, eus-fna, aih, pbc, lymph node enlargement

## Abstract

Two patients were incidentally diagnosed with intra-abdominal lymphadenopathy on imaging examinations. Although endoscopic ultrasound-guided fine needle aspiration of these areas of lymphadenopathy was performed, their causes remained undetermined. Neither patients had abnormal hepatic enzyme levels at the time lymphadenopathy was detected, but they developed hepatitis 20 months and five months later, respectively. The laboratory data and/or histopathological findings suggested primary biliary cholangitis/cirrhosis (PBC) and autoimmune hepatitis (AIH), respectively. These two patients were each started on appropriate treatment (ursodeoxycholic acid or prednisolone, respectively), their hepatitis ameliorated, and the hepatic enzyme levels recovered to within the normal ranges. These patients’ clinical courses suggest that their lymphadenopathy was associated with PBC or AIH and appeared before the causative hepatitis became clinically apparent. We should consider the possibility of latent autoimmune hepatic diseases in cases with cryptogenic intra-abdominal lymphadenopathy even if there is no clinically apparent hepatitis.

## Introduction

Lymphadenopathy is defined as lymph nodes that are abnormal in size or consistency [1]. Although lymphadenopathy is benign and self-limited in most patients [1], the presence of lymphadenopathy may lead to a diagnosis of malignant, infectious, or inflammatory diseases, such as metastatic cancer, lymphoma, tuberculosis, and amyloidosis [2-4]. Abdominal lymphadenopathy, especially perihepatic lymphadenopathy, is frequently present in chronic liver diseases other than malignant neoplastic diseases, including chronic viral hepatitis, autoimmune hepatitis (AIH), primary biliary cholangitis/cirrhosis (PBC), and primary sclerosing cholangitis (PSC) [5-13]. In these chronic hepatitis, the size and morphological changes of lymph nodes sometimes reflect the inflammatory activities of the hepatitis [5-8,13,14]. In general, a biopsy is an appropriate procedure for the evaluation and differential diagnosis of unexplained lymphadenopathy, and endoscopic ultrasound-guided fine needle aspiration (EUS-FNA) has high diagnostic accuracy [1]. However, there are many cases of lymphadenopathy with benign or inflammatory changes on cytology [2-4], and they are diagnosed as benign or inflammatory changes, with unknown causes for the lymph node enlargement. Furthermore, there is no literature about the follow-up of cases of benign and unknown causes of abdominal lymphadenopathy, and there is no literature on the relationship between lymphadenopathy of unknown cause and the potential for autoimmune hepatic diseases.

In this case report, two cases of autoimmune hepatic diseases (case 1: PBC and case 2: AIH) are presented. Both cases had lymphadenopathy with benign or inflammatory changes on EUS-FNA and no apparent hepatic disorders when the lymphadenopathy was found. However, each case developed a hepatic disorder after the detection of the lymphadenopathy, and PBC and AIH, respectively, were finally diagnosed.

## Case presentation

Two cases with cryptogenic intra-abdominal lymphadenopathy were detected. However, these cases were diagnosed with IAH after that. We should consider the possibility of latent autoimmune hepatic diseases in cases with cryptogenic intra-abdominal lymphadenopathy even if there is no clinically apparent hepatitis.

Case 1

**Figure 1:**
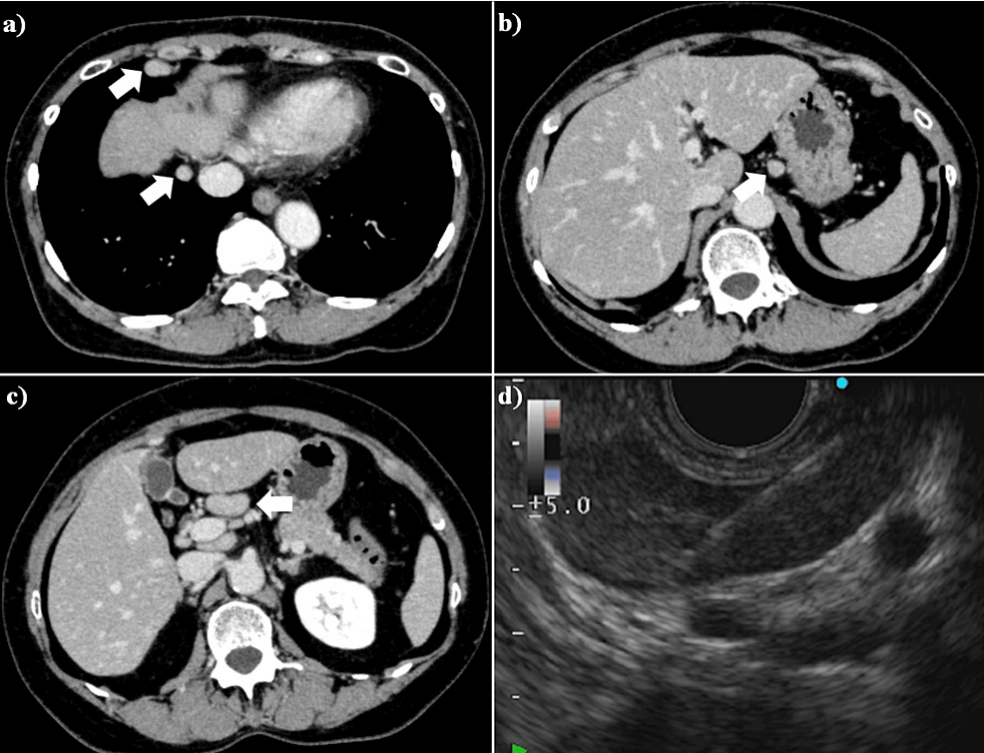
Computed tomography (CT) and endoscopic ultrasound (EUS) of the case 1 patient. Computed tomography (CT) shows the lymphadenopathy (arrows) affecting the a) supradiaphragmatic lymph nodes, b) gastric regional lymph node, and c) common hepatic arterial lymph node. d) Endoscopic ultrasound (EUS) shows lymphadenopathy as a hypoechoic mass lesion adjacent to the common hepatic artery. EUS-guided fine needle aspiration (EUS-FNA) of the lymphadenopathy is performed.

**Figure 2:**
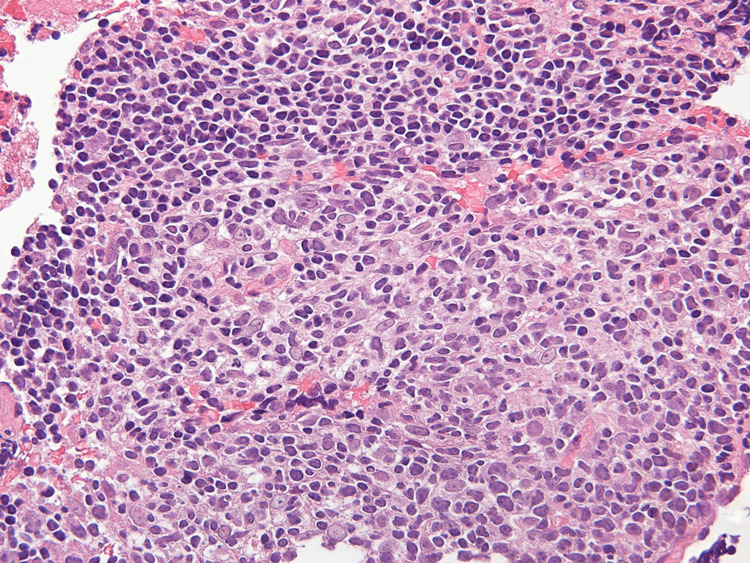
Histopathological findings of the EUS-FNA specimen from the lymphadenopathy of the common hepatic arterial lymph node with hematoxylin and eosin staining, high-power view (×40). No findings of malignant cells or granuloma formation are seen.

**Figure 3:**
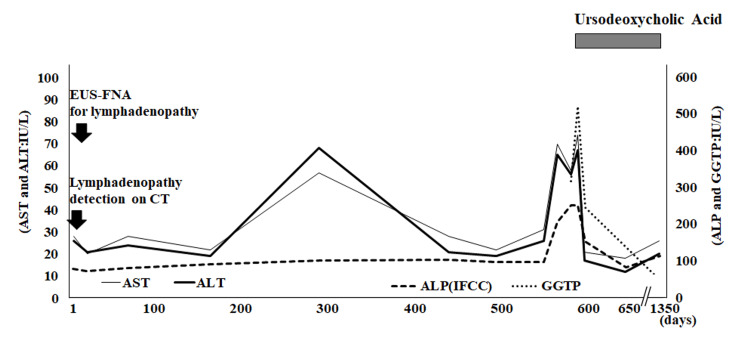
Clinical course of the case 1 patient. The gray bar indicates the treatment of ursodeoxycholic acid 600 mg/day. AST, aspartate aminotransferase; ALT, alanine aminotransferase; ALP, alkaline phosphatase; GGTP, g-glutamyltransferase

A 60-year-old woman was referred to our hospital with a chief complaint of intra-abdominal lymphadenopathy. The lymphadenopathy was first detected by ultrasonography at a health check. Her chest and abdominal computed tomography (CT) showed lymphadenopathy of the supradiaphragmatic lymph node, gastric regional lymph node, and common hepatic arterial lymph node (Fig. 1a-1c). She had no symptoms and no fever. She had no history of animal or insect exposures. On physical examination, no lymphadenopathy was found. The laboratory work-up for lymphadenopathy showed no abnormal values, except for mild elevation of the lactate dehydrogenase (LDH) level (Table 1). EUS-FNA of the common hepatic arterial lymph node swelling was performed through the trans-gastric approach using a 22-gauge needle (Expect®, Boston Scientific Co. Ltd., Marlborough, MA) (Fig. 1d). The microscopic examination did not show malignant cells or granuloma formation (Fig. 2), with no suggestion of a particular etiology. The lymphadenopathy was diagnosed as benign, with cause unknown. The patient was periodically followed up for lymphadenopathy. About 300 days the after detection of lymphadenopathy, her laboratory data showed elevations of aspartate aminotransferase (AST) and alanine aminotransferase (ALT) level, but the alkaline phosphatase (ALP) level remained within the normal range (Fig. 3). The AST and ALT elevations were transient and returned to the normal ranges spontaneously. The patient did not have any symptoms or complaints, and there was no change in the lymphadenopathy (data not shown).

**Table 1:** Laboratory data of the lymphadenopathy work-up for cases 1 and 2 CBC, complete blood count; WBC, white blood cells; RBC, red blood cells; PT, prothrombin time; PT-INR, prothrombin time-international normalized ratio; APTT, activated partial thromboplastin time; TP, total protein; Alb, albumin; AST, aspartate aminotransferase; ALT, alanine aminotransferase; LDH, lactate dehydrogenase; ALP, alkaline phosphatase; g-GT, g-glutamyltransferase; T-Bil, total bilirubin; D-Bil, direct bilirubin; ChE, cholinesterase; T-Chol, total cholesterol; BUN, blood urea nitrogen; UA, uric acid; Crea, creatinine; CRP, c-reactive protein; IL-2R, interleukin-2 receptor; HBsAg; hepatitis B surface antigen; COI, cut-off index; HCVAb, anti-hepatitis C virus antibody; HIV, human immunodeficiency virus; ND, no data; IFCC: International Federation of Clinical Chemistry; JSCC: Japan Society of Clinical Chemistry #1: Serum ALP levels measured using the IFCC method could be calculated as 0.34 times the ALP levels measured using the JSCC method.

	Reference	Case 1	Case 2
CBC			
WBC (/ml)	3900-9800	7600	5100
RBC (/ml)	427-570	477 × 10^4^	501 × 10^4^
Hemoglobin (g/dl)	13.5-17.6	14.8	14.2
Hematocrit (%)	39.8-51.8	41.0	43.4
Platelets (/ml)	130-369	24.3 × 10^4^	34.8 × 10^4^
Coagulation			
PT (%)	70-130	108	114
PT-INR	0.8-1.2	0.96	0.93
APTT (sec.)	25-45	29.2	31.4
Chemistry			
TP (g/dl)	6.5-8.5	7.7	7.6
Alb (g/dl)	4.1-5.3	4.3	4.1
AST (IU/L)	10-35	28	24
ALT (IU/L)	10-35	26	39
LDH (IU/L) (IFCC)	124-222	234	166
ALP (IU/L) (IFCC)	72-113	79^#1^	125
g-GT (IU/L)	8-60	ND	87
T-Bil (mg/dl)	0.2-1.3	1.6	0.5
D-Bil (mg/dl)	0.1-0.5	0.1	0.1
ChE (IU/L)	229-520	291	375
T-Chol (mg/dl)	150-219	181	155
BUN (mg/dl)	9.6- 22.0	9.6	11.0
UA (mg/dl)	2.0-6.9		
Crea (mg/dl)	0.50-1.10	0.64	0.55
CRP (mg/dl)	0.00-0.30	0.05	0.18
IL-2R（IU/mL）	145-519	128	909
Viral markers			
HBsAg (COI)	0.00-0.99	0.10	0.40
HCVAb (COI)	0.0-0.9	0.1	0.0
HIV		(-)	(-)

On day 579, the patient again showed elevations of AST and ALT, along with simultaneous elevations of ALP and gamma-glutamyltransferase (g-GT); the hepatic injury was associated with the elevation of immunoglobulin M (IgM), and anti-mitochondrial antibody type 2 (AMA-M2) was positive (Table 2). Primary biliary cholangitis (PBC) was suspected as the cause of the hepatitis. The patient refused a liver biopsy, and ursodeoxycholic acid 600 mg/day was started for the hepatitis with the patient’s consent. After the start of ursodeoxycholic acid treatment, the hepatic inflammation improved, with normalization of the hepatobiliary enzyme levels, and PBC was finally diagnosed. Now, about two years later, the hepatobiliary enzyme levels remain in the normal ranges on ursodeoxycholic acid 600 mg/day (Fig. 3). With regard to the lymphadenopathy, there was a slight volume reduction on imaging after the hepatobiliary enzyme levels normalized with ursodeoxycholic acid treatment (data not shown).

Case 2

**Figure 4:**
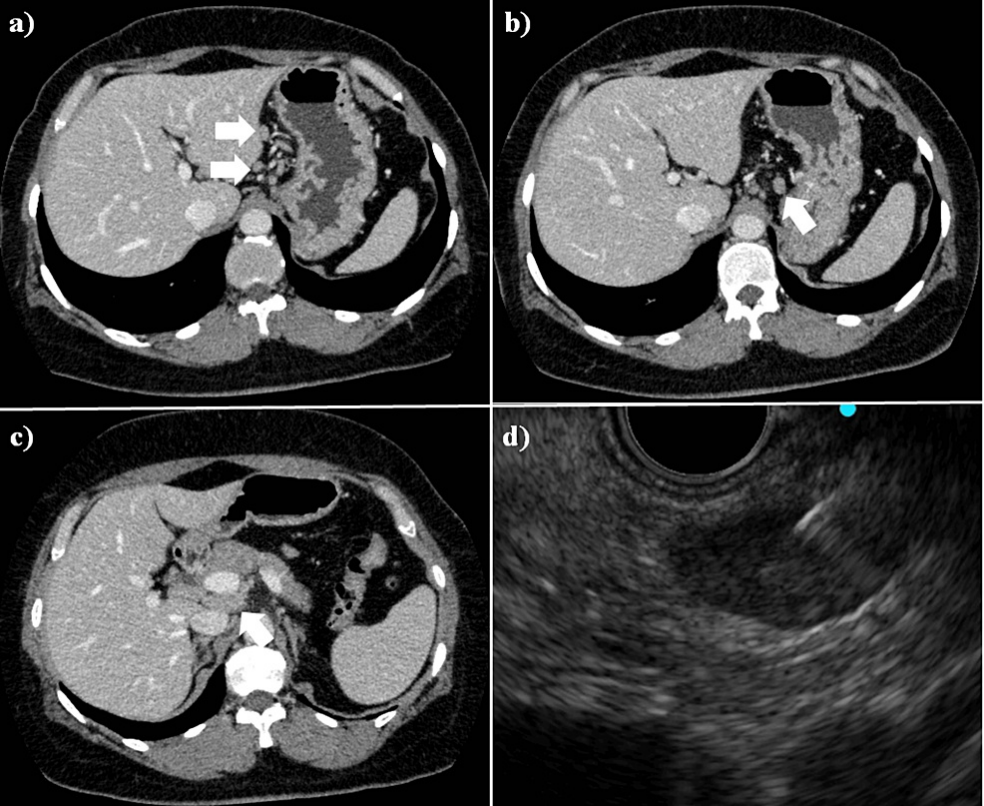
Computed tomography (CT) and endoscopic ultrasound (EUS) of the case 2 patient. Computed tomography (CT) shows the lymphadenopathy (arrows) affecting the (a and b) gastric regional lymph nodes and the c) hepatoduodenal ligament lymph node. d) Endoscopic ultrasound (EUS) shows lymphadenopathy as a hypoechoic mass lesion in the hepatoduodenal ligament. EUS-FNA of the lymphadenopathy is performed.

**Figure 5:**
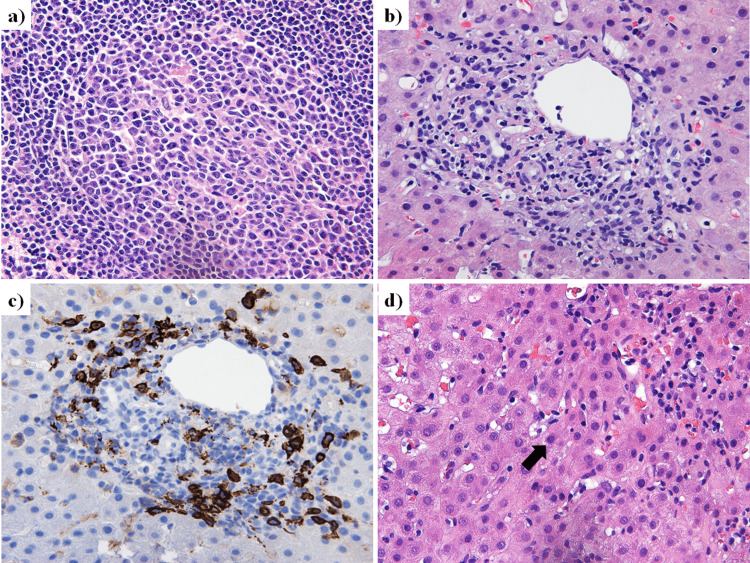
Histopathological findings of the endoscopic ultrasound-guided fine needle aspiration (EUS-FNA) specimen from the lymphadenopathy in the hepatoduodenal ligament lymph node a) and echo-guided percutaneous liver biopsy specimen b) with hematoxylin and eosin staining (high-power view: ×40). a) No findings of malignant cells or granuloma formation are seen. b) A high-power view (×40) of a liver specimen shows interface hepatitis. c) Histopathological findings of a liver specimen with cluster of differentiation 38 (CD38) immunohistochemical staining shows infiltration of plasma cells in the enlarged portal area (high-power view: ×40). c) Emperipolesis (arrow) is detected within hepatocyte cytoplasm (high-power view: ×40).

A 43-year-old woman was referred to our hospital with a chief complaint of continuous back pain for one month. She had a check-up with abdominal CT, and it showed lymphadenopathy of the gastric regional lymph node and the common hepatic arterial lymph node (Fig. 4a-4c). She had no fever and no history of animal or insect exposures. On physical examination, no lymphadenopathy was found. The laboratory work-up for lymphadenopathy showed mild elevations of ALT, ALP, and g-GT levels. Simultaneously, the value of IL-2R increased (Table 1). EUS-FNA of the common hepatic arterial lymph node was performed through a transgastric approach using a 19-gauge needle (Expect®, Boston Scientific Co. Ltd.) (Fig. 4d). Microscopic examination did not show malignant cells or granuloma formation (Fig. 5a); no particular etiology was suggested. Thus, the lymphadenopathy was diagnosed as benign, with unknown cause. The abdominal lymphadenopathy was not followed up further.

**Figure 6:**
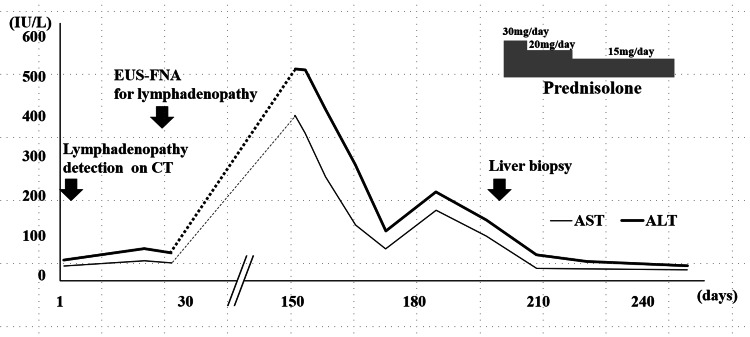
Clinical course of the case 2 patient. The gray bar indicates prednisolone treatment. Broken lines indicate no-follow up period. AST, aspartate aminotransferase; ALT, alanine aminotransferase

However, the patient was referred to our hospital with a chief complaint of general fatigue 150 days after the detection of the lymphadenopathy (Fig. 6), and her laboratory data at that time showed elevations to about ≥10-fold the normal upper limit values of AST and ALT (Table 2). Meanwhile, ALP and g-GTP showed mildly elevated and normal levels, respectively. In addition to the abnormal elevations of AST and ALT, immunoglobulin G (IgG) was elevated, and both anti-nuclear antibody (ANA) and anti-smooth muscle antibody were positive (Table 2). The elevations of AST and ALT decreased spontaneously after a peak on the 150th day, but they then increased again (Fig. 6). Autoimmune hepatitis was suspected because of the hepatic enzyme abnormalities and the positive auto-antibodies, and echo-guided percutaneous liver biopsy was performed. The histopathological findings of the liver specimen showed interface hepatitis, infiltration of plasma cells in the enlarged portal area, and emperipolesis (Fig. 6d). According to the revised criteria for diagnosis proposed by the International Autoimmune Hepatitis Group (14), the AIH score of this case was calculated to be 17, defined as AIH.

**Table 2:** Laboratory data at the time of hepatitis onset for cases 1 and 2 CBC, complete blood count; WBC, white blood cells; RBC, red blood cells; PT, prothrombin time; PT-INR, prothrombin time-international normalized ratio; APTT, activated partial thromboplastin time; TP, total protein; Alb, albumin; AST, aspartate aminotransferase; ALT, alanine aminotransferase; LDH, lactate dehydrogenase; ALP, alkaline phosphatase; g-GT, g-glutamyltransferase; T-Bil, total bilirubin; D-Bil, direct bilirubin; ChE, cholinesterase; T-Chol, total cholesterol; BUN, blood urea nitrogen; UA, uric acid; Crea, creatinine; CRP, c-reactive protein; IL-2R, interleukin-2 receptor; HBsAg; hepatitis B surface antigen; COI, cut-off index; HCVAb, anti-hepatitis C virus antibody; HBsAb, immunoglobulin G antibody to the hepatitis B surface antigen; IgG-HBc, immunoglobulin G antibody to the hepatitis B core antigen; IgM-HBc, immunoglobulin M antibody to the hepatitis B core antigen; IgM-HSV, immunoglobulin M antibody to herpes simplex virus; IgM-CMV, immunoglobulin M antibody to cytomegalovirus; IgM-EBV, immunoglobulin M antibody to Epstein-Barr virus; EBNA, Epstein-Barr virus-nuclear antibody; IgM-HAV, immunoglobulin M antibody to hepatitis A virus; IgA-HEV, immunoglobulin A antibody to hepatitis E virus; HCVRNA, hepatitis C virus ribonucleic acid; IgG, immunoglobulin G; IgA, immunoglobulin A; IgM, immunoglobulin M; ANA, anti-nuclear antibody; AMAM2, anti-mitochondrial antibody M2 subtype; ASMA, anti-smooth muscle antibody; ND, no data; IFCC: International Federation of Clinical Chemistry; JSCC: Japan Society of Clinical Chemistry #1: Serum ALP levels measured using the IFCC method could be calculated as 0.34 times the ALP levels measured using the JSCC method.

	Reference	Case 1	Case 2
CBC			
WBC (/ml)	3900-9800	7300	5500
RBC (/ml)	427-570	428 × 10^4^	455 × 10^4^
Hemoglobin (g/dl)	13.5-17.6	13.3	12.7
Hematocrit (%)	39.8-51.8	38.6	39.0
Platelets (/ml)	130-369	19.8 × 10^4^	27.9 × 10^4^
Coagulation			
PT (%)	70-130	90	92
PT-INR	0.8-1.2	1.06	1.04
APTT (sec.)	25-45	31.4	30.3
Chemistry			
TP (g/dl)	6.5-8.5	7.2	7.1
Alb (g/dl)	4.1-5.3	3.4	3.6
AST (IU/L)	10-35	70	339
ALT (IU/L)	10-35	65	458
LDH (IU/L) (IFCC)	124-222	316	209
ALP (IU/L) (IFCC)	72-113	204^#1^	128
g-GT (IU/L)	8-60	316	56
T-Bil (mg/dl)	0.2-1.3	1.1	4.4
D-Bil (mg/dl)	0.1-0.5	0.1	3.2
ChE (IU/L)	229-520	206	243
T-Chol (mg/dl)	150-219	142	155
BUN (mg/dl)	9.6- 22.0	5.1	10.2
UA (mg/dl)	2.0-6.9	ND	ND
Crea (mg/dl)	0.50-1.10	0.62	0.53
CRP (mg/dl)	0.00-0.30	3.90	0.06
Viral markers			
HBsAg (COI)	0.00-0.99	0.43	0.39
HCVAb (COI)	0.0-0.9	0.0	0.0
HBsAb (mIU/ml)	0-10	2	2
IgG-HBc		(-)	(-)
IgM-HBc		ND	(-)
IgM-HSV	0.00-0.79	ND	0.80
IgM-CMV (S/CO)	0.00-0.85	ND	0.32
IgM-EBV	0.0-9.9	ND	10
EBNA	0.0-9.9	ND	40
IgM-HAV (S/CO)	0.0-0.8	ND	0.18
IgA-HEV		ND	(-)
HCVRNA (logIU/ml)	0.0-1.1	ND	<1.2
Serology			
IgG (mg/dl)	870-1700	1613	1789
IgA (mg/dl)	110-410	213	280
IgM (mg/dl)	35-220	521	83
Ferritin (ng/ml)	6.2-138	ND	105
IL-2R (U/dl)	145-519	2782	1024
ANA	0-39	40	40
AMAM2	0.0-6.9	261	20
ASMA	0.0-19.9	<20	80

Therefore, the diagnosis of this case was AIH. The patient was started on treatment with prednisolone at a dose of 30 mg/day. After the start of prednisolone treatment, the hepatitis ameliorated, and the AST and ALT levels normalized (Fig. 6). Now, three months later, the hepatobiliary enzyme levels remain in the normal range on treatment with a maintenance dose of prednisolone (5 mg/day). With regard to the lymphadenopathy, there was a slight volume reduction on imaging after the normalization of the hepatic enzyme levels with prednisolone treatment (data not shown).

## Discussion

Autoimmune hepatic diseases, including AIH and PBC, are forms of chronic active hepatitis, and diagnosing the precise moment of disease onset is difficult, especially in acute-onset AIH [15], because of the characteristic disease latency during the early course. Since both AIH and PBC cause persistent inflammatory damage to liver tissues, the patient eventually develops cirrhosis and hepatic failure without appropriate treatment [16,17]. Therefore, early diagnosis and start of adequate treatment are necessary for AIH and PBC. In the present two cases, lymphadenopathy involving intra-abdominal perihepatic lymph node swelling was detected first in the disease course. However, the possibility of autoimmune hepatic disease could not be suspected at the time of lymphadenopathy detection because there were no abnormal elevations of hepatobiliary enzyme levels. Although some reports described the utility of intra-abdominal lymph node biopsy for differential diagnosis [2-4], there has been no literature about the association between autoimmune hepatic disease and lymphadenopathy of unknown etiology. If any tests for related autoantibodies, for example, ANA, AMA, or ASMA, had been performed earlier, these autoimmune hepatic diseases might have been diagnosed earlier. Therefore, we should consider the possibility of autoimmune hepatic diseases, even if hepatobiliary enzyme levels are normal, when intra-abdominal lymphadenopathy is found with no apparent causative disease. In addition, the patient with intra-abdominal cryptogenic lymphadenopathy and no apparent characteristics of hepatitis requires regular follow-up for the early detection of a possible future onset of hepatitis. Because reactive lymphadenopathies often result in diagnostic ambiguity too, we should consider the possibility of autoimmune hepatic disease with that.

In both cases, IL-2R levels were elevated. Their elevations did not reflect the existence of malignant lymphoma because of the tumor cells’ negative pathological findings on lymph node biopsy and the morphologically unchanged lymph nodes from lymphadenopathy detection to the onset of hepatitis (data not shown). IL-2R is not currently included in the diagnostic criteria for AIH and PBC [14,18], but both AIH and PBC have been reported to show significantly higher levels of IL-2R [19,20], and chronic liver disease patients independent of the underlying etiology also show significantly elevated serum IL-2R levels [21]. Since IL-2R is not only elevated in neoplastic disorders but also in autoimmune diseases, for example, systemic lupus erythematosus and rheumatoid arthritis [22], the IL2-R level is considered to reflect disease activity in inflammatory disorders. In the present cases, the elevated IL-2R levels might have reflected the inflammatory activities of AIH and PBC. In addition, an undetected hematological malignancy with intra-abdominal cryptogenic lymphadenopathy and IL-2R elevation may imply some kind of potential autoimmune hepatic diseases, even if there are no apparent hepatic enzyme abnormalities. However, we should interpret the value of the IL-2R level carefully, because it is reported to be normal in chronic non-active hepatitis [23].

In case 1, lymphadenopathy was detected in a supradiaphragmatic lymph node, in addition to the intra-abdominal lymph nodes. However, this supradiaphragmatic lymphadenopathy did not increase in size during the follow-up period, similar to that of the other intra-abdominal lymphadenopathy. In some case reports, systemic lymph node enlargement has been described in PBC cases [24,25]. Thus, the supradiaphragmatic lymphadenopathy in case 1 was assumed to be associated with PBC, and the lymphadenopathy in the intra-abdominal cavity may reflect the progression of PBC. Therefore, careful follow-up of PBC cases with extra-abdominal lymph node enlargement, as in the present case, is needed, because these PBC cases might have severe hepatic injury and hepatic dysfunction.

There was only slight volume reduction of the lymphadenopathy after normalization of hepatic function in the present cases. Fujii et al. reported that the size of markedly enlarged hepatoduodenal ligament lymph nodes decreased after improved hepatobiliary enzymes in a case of AIH [20]. However, a negative association between lymph node size and hepatocellular function has also been reported [5], and Lyttkens et al. reported that lymph node size decreased slightly during two years of treatment in PBC cases [6]. Since the changes in lymph node size were accompanied by changes of immunoreactivity [5], the enlarged lymph nodes may reflect the continuing immunoreactivity in the present cases despite the normalization of hepatobiliary enzymes by effective treatment. If lymph nodes disappeared or their size reduced with treatment, our suggested association between precursor lymphadenopathy and autoimmune liver diseases may augment.

This case report is consisted in two real-world cases only, and more cases are necessary to analyze in detail in the future.

## Conclusions

Two cases of autoimmune hepatic disorders (AIH and PBC) that had shown lymphadenopathy before the hepatitis became clinically apparent were presented. The lymphadenopathy did not include malignant cells or specific pathological findings, but hepatobiliary enzyme levels increased after lymphadenopathy detection, and the two cases were finally diagnosed as having autoimmune hepatic diseases concomitant with lymphadenopathy.

We should consider the possibility of latent autoimmune hepatic diseases in cases with lymphadenopathy, especially when intra-abdominal, which have unknown causes, even when hepatic disease is not apparent. Checking autoimmune hepatic disease-related autoantibodies or immunoglobulins is desirable for the early diagnosis and treatment of cases of cryptogenic intra-abdominal lymphadenopathy.
